# Understanding Intra-Species and Inter-Species Prion Conversion and Zoonotic Potential Using Protein Misfolding Cyclic Amplification

**DOI:** 10.3389/fnagi.2021.716452

**Published:** 2021-08-03

**Authors:** Alexander H. Peden, Suzanne Suleiman, Marcelo A. Barria

**Affiliations:** National CJD Research & Surveillance Unit, Centre for Clinical Brain Sciences, Deanery of Clinical Medicine, The University of Edinburgh, Edinburgh, United Kingdom

**Keywords:** neurodegenarative disease, prion, CJD (Creutzfeldt-Jakob disease), protein misfolding, *in vitro* aggregation assays, surveillance, zoonosis, prion diseases

## Abstract

Prion diseases are fatal neurodegenerative disorders that affect humans and animals, and can also be transmitted from animals to humans. A fundamental event in prion disease pathogenesis is the conversion of normal host prion protein (PrP^C^) to a disease-associated misfolded form (PrP^Sc^). Whether or not an animal prion disease can infect humans cannot be determined *a priori*. There is a consensus that classical bovine spongiform encephalopathy (C-type BSE) in cattle transmits to humans, and that classical sheep scrapie is of little or no risk to human health. However, the zoonotic potential of more recently identified animal prion diseases, such as atypical scrapie, H-type and L-type BSE and chronic wasting disease (CWD) in cervids, remains an open question. Important components of the zoonotic barrier are (i) physiological differences between humans and the animal in question, (ii) amino acid sequence differences of the animal and human PrP^C^, and (iii) the animal prion strain, enciphered in the conformation of PrP^Sc^. Historically, the direct inoculation of experimental animals has provided essential information on the transmissibility and compatibility of prion strains. More recently, cell-free molecular conversion assays have been used to examine the molecular compatibility on prion replication and zoonotic potential. One such assay is Protein Misfolding Cyclic Amplification (PMCA), in which a small amount of infected tissue homogenate, containing PrP^Sc^, is added as a seed to an excess of normal tissue homogenate containing PrP^C^, and prion conversion is accelerated by cycles of incubation and ultrasonication. PMCA has been used to measure the molecular feasibility of prion transmission in a range of scenarios using genotypically homologous and heterologous combinations of PrP^Sc^ seed and PrP^C^ substrate. Furthermore, this method can be used to speculate on the molecular profile of PrP^Sc^ that might arise from a zoonotic transmission. We discuss the experimental approaches that have been used to model both the intra- and inter-species molecular compatibility of prions, and the factors affecting PrP^c^ to PrP^Sc^ conversion and zoonotic potential. We conclude that cell-free prion protein conversion assays, especially PMCA, are useful, rapid and low-cost approaches for elucidating the mechanisms of prion propagation and assessing the risk of animal prions to humans.

## Overview

### Prion

Prions – *Proteaceous infectious particles*- are self-propagating structures identified as the major or sole cause of prion diseases, which are lethal neurodegenerative disorders that affect animals and humans. The pathogenesis of prion diseases is strongly believed to be related to protein misfolding. The normal prion protein, PrP^C^, encoded by *PRNP*, is a highly conserved cell surface glycoprotein. It is thought to have a number of physiological roles in both the central and peripheral nervous system and may serve as a regulatory protein in cell proliferation and differentiation (Halliez et al., 2014; Wulf et al., 2017). In prion disorders, PrP^C^ is believed to change its conformation and become misfolded. The abnormal misfolded version, PrP^Sc^, is partly resistant to proteinase K and can self-reproduce by causing PrP^C^ to convert to its abnormal isoform. The resultant misfolded molecules form multi-chain fibrillary/amyloid aggregates. Over months to several years *in vivo*, these structures accumulate in brain and nerve tissue, triggering cell death and the neurodegeneration associated with prion diseases (Prusiner, 1982; Ironside et al., 2014; Love et al., 2015).

### Animal and Human Prion Diseases

Animal prion diseases include bovine spongiform encephalopathy (BSE) in cattle, scrapie in sheep and goats, transmissible mink encephalopathy (TME) and chronic wasting disease (CWD) in several cervid species, including, elk, moose, and reindeer or caribou (Sigurdson and Aguzzi, 2007). More recently, a novel type of prion disease was reported in dromedary camels (Babelhadj et al., 2018). In humans, prion diseases are clinically and pathologically heterogeneous, and can present as either (1) spontaneous disorders, e.g., sporadic CJD (sCJD) and variably protease-sensitive prionopathy (VPSPr); (2) genetic disorders, including Gerstmann-Sträussler-Scheinker disease (GSS) and genetic CJD (gCJD) (Richt and Hall, 2008); (3) and acquired/transmissible forms, such as variant CJD (vCJD), iatrogenic CJD (iCJD) and kuru. The transmissible neurodegenerative diseases caused by prions are also known collectively as “transmissible spongiform encephalopathies” or TSEs.

### Prion Strains

Currently, the protein-only hypothesis remains the most accepted paradigm in prion research. In this model, prions are transmissible particles that are devoid of nucleic acid and seem to be composed entirely of a modified disease-associated form of the prion protein (PrP^Sc^) (Griffith, 1967; Prusiner, 1982). In this model PrP^Sc^ self-propagates by conversion of PrP^C^ and is able to infect new individuals and sometimes, as in BSE, is able to transmit to a different species. However, PrP^Sc^ still resembles other conventional infectious agents by expressing characteristic strain variations; i.e., a particular prion strain can encode one of various specific disease phenotypes that are maintained after inoculation and serial passage in experimental animals. It was initially assumed that the strain phenomenon might be a function of the PrP^C^ primary structure of the host. However, different prion strains were also identified in genetically identical mice suggesting that strain variation is not simply dependent on amino acid similarities between donor and recipient (Collinge and Clarke, 2007). According to the protein-only hypothesis the strain-specific properties are enciphered in the various conformations that PrP^Sc^ can adopt and are maintained when PrP^Sc^ replicates through conversion of PrP^C^ (Griffith, 1967; Prusiner, 1982).

Prion diversity has historically been investigated by experimental transmission studies in animal models, where several criteria, including the length of time between inoculation and appearance of clinical signs, the type of clinical signs, neuropathological changes (score profile) and biophysical measures can be measured (Brandner and Jaunmuktane, 2017; Igel-Egalon et al., 2018). The classical way of biochemically classifying prion diversity is by PrP^Sc^ proteolysis and studying the biochemical profile of the protease resistant core on western blot analysis. Three major bands are usually observed upon cleavage with proteinase K, corresponding to un-, mono- and di-glycosylated forms of PrP. The size and relative abundance of these three forms is usually characteristic of a particular prion variant, referred to as the PrP^res^ subtype (Head and Ironside, 2012). In the classification of human prion disease subtypes using the nomenclature of Parchi (Parchi et al., 2012), unglycosylated bands at 21 or 19 kDa are associated with PrP^Sc^ types 1 and 2, respectively. In addition, a predominance of either the mono- or di-glycosylated band is assigned as type A and B, respectively. All sporadic CJD cases are of the ‘A’ glycoform and are either type 1 or 2, whereas variant CJD is associated with type 2B. These differences in fragment size and glycosylation ratios are believed to be associated with different PrP^Sc^ conformations, which are in turn associated with different prion strains.

Polymorphic differences clearly play a key role in influencing prion strains in human prion disease. It is well-recognized that codon 129 of the prion protein gene *PRNP*, which has either methionine (M) or valine (V), correlates with the molecular typing and prion strain characteristics (Love et al., 2015). The strain phenomenon is observed in sporadic forms of prion disease. Sporadic CJD Type 1 subtype is favored in individuals who are methionine homozygous (MM) at polymorphic codon 129 of *PRNP*, whereas valine homozygosity at this codon favors Type 2. MV heterozygous individuals with sCJD are more evenly split between Type 1 and Type 2 (Parchi et al., 1999). The association of sCJD subtypes with strains was supported by transmission studies to transgenic mice expressing human *PRNP* (TgHu) with the three *PRNP* codon 129 genotypes (MM, MV, and VV), which suggested the existence of four prion strains (M1, M2, V1, and V2), and a fifth strain corresponding to a thalamic sub-subtype, MM2T, also known as sporadic fatal insomnia (Bishop et al., 2010; Moda et al., 2012). However, the example of sCJD subtypes alludes to the existence of an overlap between prion strains, with the commonly observed co-existence of more than one PrP^Sc^ subtype within individual sCJD patients (Polymenidou et al., 2005; Uro-Coste et al., 2008; Cali et al., 2009; Parchi et al., 2009).

### Conformation Selection Theory

In light of evidence that prion strains may not be absolutely discreet entities, Collinge and Clarke (2007) proposed a conformation selection theory in which a prion strain is thought to be comprised of not just one type of PrP^Sc^ molecular assembly, but instead a range or cloud of assemblies with different tertiary and quaternary structures. In this model, a subset of assemblies within this cloud may be selected for replication in a new individual of the same species with an alternate *PRNP* genotype, or in a new species. The transmission will occur more readily if a significant subset of conformers of the prion strain can successfully replicate in the new host, and this will be determined in part by the compatibility of the PrP sequence.

### Development of *in vitro* Models as Versatile and Reproducible Alternatives to Investigate Prion Diseases

Evaluation of transmission barriers using the *in vivo* models requires the use of large numbers of experimental animals. In addition, despite the development of the transgenic animal models, the speed with which animal research can be conducted has been hindered by long incubation periods, especially when serial passage is being used to evaluate prion strain stabilization or adaptation. Therefore, there has been an urgent need to develop alternative, quicker, more cost-efficient and reliable *in vitro* models.

The first *in vitro* cell-free system for modeling prion conversion was demonstrated by Kocisko et al. (1994). They showed that the radiolabeled recombinant hamster PrP^C^ was able to selectively convert to its PK-resistant PrP^Sc^ isoform in the presence of unlabeled hamster PrP^Sc^. This conversion occurred even after de-glycosylation of PrP^Sc^ or transient denaturation with a chaotropic agent (GdnHCl) (Kocisko et al., 1994).

### Protein Misfolding Cyclic Amplification (PMCA)

**FIGURE 1:**
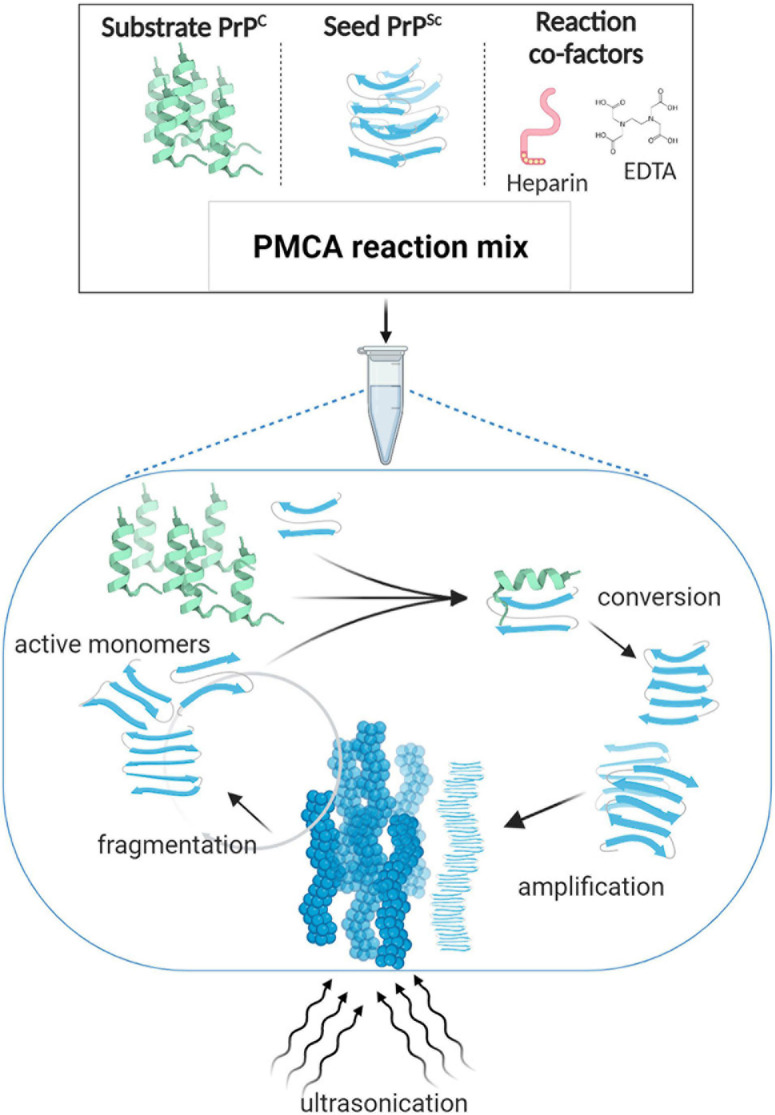
Protein misfolding cyclic amplification (PMCA). PMCA reaction mix is composed of minute amounts of the misfolded abnormal β sheet-rich prion protein (seed PrP^Sc^) and a large excess of the normal α-helix prion protein (substrate PrP^C^), in addition to reaction cofactors such as ethylenediaminetetraacetic acid (EDTA) and heparin as reported for some of the established protocols (Barria et al., 2014b, 2018a; Ritchie et al., 2017). The PMCA reaction mimics the *in vivo* PrP conversion; i.e., according to the template-directed model of propagation, when incubated at 37°C, PrP^Sc^ interacts with PrP^C^ and thereby alters its conformation. The newly formed misfolded subunits grow and aggregate to form large fibrillary structures. Ultrasonication is applied to break down the misfolded aggregates into active converting “seeds” that lead the reaction again in a cyclic manner. By the end of one 48-h round of PMCA, most PrP^C^ molecules are converted into its misfolded form, enabling their subsequent detection by western blotting.

**FIGURE 2:**
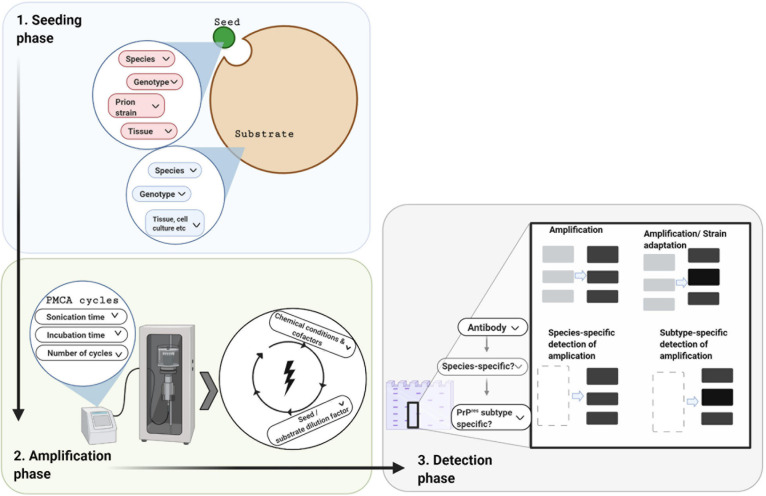
Protein misfolding cyclic amplification and its three technical phases. In designing a PMCA experiment there are three phases to consider, which each involve decisions depending on the research question and resources available. In the seeding phase, a minute amount of PrP^Sc^ seed (small green circle) is mixed with an excess of PrP^C^ substrate (large brown circle). For both these components, the species, *PRNP* genotype, and tissue source need to be chosen. In addition, the prion strain of the seed needs to be selected. In the amplification phase, cofactors may be added to the above mixture which is then subjected to cycles of incubation and sonication. Choices are required for the physical parameters (e.g., cycle time, incubation versus sonication time per cycle, number of cycles, and amplitude of ultra-sonication). In the detection phase, the amplified product is quantified and/or characterized, usually by western blotting following limited protease digestion. Cross-species amplification can be confirmed with species-specific detection antibodies, whereas alterations in the PrP^res^ molecular profile can be confirmed with subtype-specific antibodies.

A few years later, Saborio et al., 2001 successfully established a procedure that applied cycles of incubation and sonication to efficiently accelerate the conversion of PrP^C^ in a hamster brain homogenate by hamster PrP^Sc^. The process was named Protein Misfolding Cyclic Amplification, or PMCA (Saborio et al., 2001; Figure 1). In the seeding phase of PMCA, an excess amount of PrP^C^ (termed the substrate) is seeded with small quantities of PrP^Sc^ (termed the seed). During the amplification phase, the mixture is incubated at 37°C and the PrP^Sc^ seed induces the generation of large PrP^Sc^ aggregates at the expense of PrP^C^ (Figure 1). PMCA thus mimics the natural propagation of PrP^Sc^ and its aggregation in the brains of TSE-infected individuals. In order to increase amplification efficiency, ultrasound power is applied periodically to break down these large misfolded aggregates and produce more of the active converting units, which accelerates the reaction in an exponential manner. By the end of one round of PMCA, most molecules are present in their misfolded conformation and PrP^Sc^ becomes detectable. In the detection phase of PMCA, the amplified product is detected, usually by western blotting following limited proteolysis to detect the protease resistant fragments of PrP^Sc^ (PrP^res^) (Figure 2). Several studies that followed the original demonstration of PMCA indicated the flexibility, fidelity and reliability of this method. PMCA was shown to reproduce the infected agent *in vitro* by serially repeating the amplification round on the diluted product (Castilla et al., 2008b, 2005). The infectivity per unit of PMCA PrP^Sc^ was comparable to that of PrP^Sc^ from the infected tissue used as the seed (Castilla et al., 2005). The serially amplified product, which becomes devoid of the original seed, was shown to hold a comparable secondary structure, electrophoretic mobility and glycosylation profile to that of the seed (Castilla et al., 2005). Moreover, strain-specific infectivity was revealed to be conserved within the amplified product when four mouse-adapted scrapie (RML, ME7, 139A, 79A), one mouse adapted BSE (310C) and four human prion disease (vCJD, sCJD MM1, sCJD MM2, sCJD VV2) PMCA-propagated strains were inoculated into experimental animals. Indeed, after serial rounds of PMCA, the PMCA-amplified product caused a comparable disease phenotype in experimental animals (incubation time, clinical signs and neuropathological profile) as the original seed (Castilla et al., 2008b; Cali et al., 2019). Thus, the characteristics of the original strain used as seed seems to be still encoded within the PMCA amplified product, providing fundamental proof that the strain characteristics, including infectivity, are preserved after *in vitro* amplification. In only a few weeks this technique can replicate PrP^Sc^ and conserve prion strain properties in a manner that recapitulates the time-consuming *in vivo* approach that might take several years.

The development of *in vitro* conversion assays, such as PMCA, opened a new era in prion research. In time, improvements in the sensitive of PMCA allowed the detection of extremely low levels of PrP^Sc^ in peripheral tissues and biological fluids in human samples (Moda et al., 2014; Bougard et al., 2016, 2018; Concha-Marambio et al., 2016; Barria et al., 2018a; Concha-Marambio et al., 2020).

## Utilization of PMCA to Investigate Homologous Molecular Compatibility Associated With Prions Conversion

As mentioned before, in humans, prion strain variation and the polymorphic codon 129 of *PRNP* are two of the major biological determinants of the clinicopathological phenotype of the disease and an individual’s susceptibility to develop prion diseases. *PRNP* polymorphism imposes its effect by altering prion strain conformation. Empirical evidence for this came from Parchi et al. (2000) who showed that codon 129 is a primary determinant of the proteinase cleavage site of PrP^Sc^ in CJD, resulting in a change in the size of the PrP proteinase resistant fragments (Parchi et al., 2000).

Although allelic variation percentages for this codon vary across different ethnic groups, homozygosity of methionine is a major risk factor in all CJD types (Deslys et al., 1998). Indeed, with the exception of one case, all clinical and pathologically confirmed vCJD cases, including three cases of secondary vCJD following blood transfusion, have had an MM genotype at codon 129. The exception is the last reported United Kingdom case that was MV at codon 129 (Mok et al., 2017). Similarly, susceptibility to sCJD is considerably higher in methionine homozygous individuals (Palmer et al., 1991). In addition, sporadic CJD patients who are codon 129 heterozygous have longer durations of disease (Pocchiari et al., 2004).

To shed more light on the role of codon 129 polymorphism on prion strain characteristics and transmissibility, Jones et al., 2007 recapitulated the transmission properties of vCJD and sCJD in two important PMCA studies. The first employed PMCA to model secondary vCJD transmission in humans (Jones et al., 2007). This study used either healthy human brain tissues or humanized transgenic mice models expressing the three possible codon 129 genotypes (MM, MV, and VV). Interestingly, vCJD propagated efficiently in both MM substrates, supporting the idea that codon 129 has a strong influence on PrP^Sc^ conversion and infectivity. Less amplification was observed in MV2 transgenic mouse brain tissues, while no amplification was observed in VV substrates, which suggested that this genotype might be a protective factor.

This study emphasized the impact of *PRNP* codon 129 genotype on susceptibility to human-to-human transmission of vCJD and indicated that MM imposes a higher risk than the other two genotypes. MV individuals still have some risk of developing vCJD and might remain asymptomatic for long periods before developing clinical disease.

The second PMCA study by Jones et al. used (MM/MV/VV) homogenates as substrates and six sCJD disease PrPSc seeds representing the six phenotypic subtypes based on subtype and codon 129 genotype: MM1/MV1, VV1, MM2 cortical, MM2 thalamic, MV2 and VV2 (Jones et al., 2008). As with vCJD, the results indicated that PMCA was an attractive model for investigating the molecular basis of phenotypic variability in sCJD. A considerable degree of compatibility in terms of the PrP^Sc^ subtype was observed between the sCJD seed and the PMCA product. Here too, codon 129 polymorphism was shown to have an impact on amplification efficiency, which reflected the relationship observed between PrP^Sc^ subtype and codon 129 genotype in sCJD patients. Successful amplification was recorded when seed and substrate codon 129 polymorphisms were matched. Remarkably, MV1 resembled MM1 as it tended to amplify better with MM substrate, whereas MV2 preferred VV substrate. Therefore, the substrate specificity of PrP^Sc^ from MV sCJD patients appeared to depend on PrP^Sc^ type.

### Iatrogenic Creutzfeldt-Jakob Disease: Molecular Approaches

Acquired forms of CJD caused by prion infection as a result of medical or surgical interventions are collectively referred as iatrogenic CJD (iCJD). Apart from secondary transmission of vCJD via blood or blood products (Peden et al., 2004; Urwin et al., 2016), iCJD also includes cases acquired by the use of contaminated medical instruments (Bernoulli et al., 1977; Will and Matthews, 1982), transplantation of contaminated corneal grafts (Duffy et al., 1974), cadaveric dura mater (DM-iCJD) (Brown et al., 2012), or treatment with contaminated cadaveric growth hormone (GH-iCJD) (Gibbs et al., 1985; Koch et al., 1985; Powell-Jackson et al., 1985; Ritchie et al., 2017). DM-iCJD and GH-iCJD comprise the majority of iCJD cases worldwide (Brown et al., 2012; Ritchie and Barria, 2021).

The *PRNP* codon 129 genotype affects susceptibility and disease phenotype in iCJD. Surveillance studies on a French cohort indicated that the majority of the GH-iCJD cases were codon 129 methionine homozygous (Delisle et al., 1993; Brandel et al., 2003). In contrast, where codon 129 data is available most United Kingdom cases of iCJD are either heterozygous (MV), or valine homozygous (VV) and only a low proportion ∼10% were homozygous for methionine (MM) (Brandel et al., 2003; Rudge et al., 2015; Ritchie et al., 2017). This suggests that original sources of contamination for the United Kingdom and French GH-iCJD epidemics were of different prion strains. The MM cases tended to occur later in United Kingdom GH-iCJD epidemic, suggesting a longer incubation period for patients with this genotype, which may indicate a genotypic barrier in these individuals to the contaminating prion strain. Alternatively, the MM cases may be the result of infection from a minor component of a mixture of contaminating strains.

Although the use of cell-free assays to model iatrogenic CJD is limited, PMCA was used in combination with other biochemical analyses to investigate the prion strain origin of the United Kingdom GH-iCJD epidemic, and the possibility that MM GH-iCJD cases might exhibit the traceback phenomenon (Ritchie et al., 2017).

### Traceback Phenomenon

The traceback phenomenon was observed in animal research studies when sCJD brain inocula of V2 strain was transmitted to humanized mice MM at *PRNP* codon 129 (Kobayashi et al., 2007). This transmission resulted in a new strain characterized by a unique disease phenotype featuring long incubation periods, and kuru-like plaques on histological examination. In addition, this strain was associated with an altered PrP^Sc^ conformation that caused the appearance of an upshifted proteinase-resistant unglycosylated band at ∼20 kDa on western blots, referred to as intermediate PrP^Sc^ (PrP^Sc^ subtype i). When this strain was inoculated on second passage into TgHu VV mice, the PrP^Sc^ appeared to regain its original conformation, resulting in type 2 PrP^Sc^ (Kobayashi et al., 2007). This property suggests that the new strain (PrP^Sc^ i) still retains within its conformational cloud some traits from the original V2 strain that are “inherited,” which means that when transmitted into new animals it can express its original strain characteristics once again (Kobayashi et al., 2009, 2010).

### PMCA Investigation of Traceback Phenomenon

Two PMCA methods have been used to investigate the traceback phenomenon. Takeuchi et al. (2016) utilized cell-PMCA (where the PrP^C^ source was from a 293F cell-lysates, overexpressing the human form of the prion protein) to amplify brain homogenate samples from two DM-iCJD cases, both MM at codon 129 in Japan. The cases considered for the study included a case of non-plaque-type DM-iCJD (np-dCJD) resembling sCJD MM1/MV1, while the other case was plaque-type DM-iCJD (p-dCJD) characterized by kuru plaques and PrP^Sc^ with an intermediate type (PrP^Sc^ subtype i). The p-dCJD PrP^Sc^ material presented molecular compatibility when a substrate homozygous for valine, not methionine, was used. The amplified material resembled the sCJD V2 strain biochemical typing. These results appear to defy the expected relationship between PMCA efficiency and the genotype compatibility of seed and substrate. The authors suggested this was evidence that plaque-type DM-iCJD was the result of transmission of the V2 CJD strain to codon 129 MM patients, and its efficient amplification in VV substrate was an indication of the traceback phenomenon.

Ritchie et al. (2017) utilized several approaches to model 21 United Kingdom cases of GH-iCJD and three cases of DM-iCJD. In particular, the authors were interested in two GH-iCJD cases homozygous for methionine at codon 129: One featured the plaque subtype and had PrP^Sc^ type i (subtype MMi), whereas the other was of type 1 and had no plaques (subtype MM1). Samples of cerebral cortex tissue from these cases were used to seed humanized transgenic mouse brain substrate carrying the codon 129 MM and VV. These two MM GH-iCJD cases gave divergent results. For the MMi case amplification was observed with the valine, not the methionine, homozygous substrate, whereas the MM1 cases failed to amplify efficiently with either substrate. More importantly, the molecular strain typing profile of the amplified product of the VV substrate seeded with MMi showed a downshifted migration of the proteinase-resistant unglycosylated form by western blotting when compared to the original material. In contrast when MM substrate was used to amplify PrP^Sc^ from the MMi case, an upshift occurred. The amplification of MMi PrP^Sc^ with VV substrate and the downshift of the molecular profile is possibly indicative of the traceback phenomenon mentioned earlier; i.e., it may provide indirect evidence that the MMi subtype results from the replication V2 strain in a genotypically mismatched methionine homozygous individual and that this subtype readily reverts to a downshifted, type 2-like molecular profile when provided with a suitable VV substrate for replication.

Both studies strongly indicated that PMCA is a rapid and reliable method for modeling the molecular mechanisms of prion transmission across genotypic barriers and the traceback phenomenon. It would appear to be possible to identify DM-iCJD cases in MM individuals that actually originated from contamination with the V2 sCJD strain, rather than the M1 strain. Additionally, Kobayashi et al. (2016) have suggested that kuru plaque pathology and PrP^Sc^ type i in codon 129 MM patients diagnosed with sCJD may actually indicate an acquired or iatrogenic etiology in these patients. Therefore, PMCA could help ascertain the acquired origin of atypical sCJD cases, without having actual evidence of iatrogenic exposure by examining the potential for PrP^Sc^ amplification using matched and mismatched humanized substrates (*PRNP* codon 129 MM or VV).

## Utilization of PMCA to Investigate Heterologous Molecular Compatibility Associated With Prions Conversion

### The Species Barrier

The species barrier refers to the combined factors that limit the ability of a pathogen from one species to cross-infect another species. Zoonotic potential refers to the likelihood this barrier might be breached to infect humans. When examining the barrier that may exist between two species for the transmission of a given prion strain, the species from which the prion strain originates can be considered to be the species of origin. The species whose threat is being assessed (e.g., humans) is the target species. Any other species through which the prion strain might pass between the species of origin and the target species is an intermediate species.

### Zoonotic Prion Disease Threats to Humans

Bovine spongiform encephalopathy is a prion disease of cattle, first recognized in 1986 in the United Kingdom, reaching epidemic proportions and then being brought under control (Hope, 2013). Dietary exposure to BSE is thought to be the cause of vCJD in humans (Will et al., 1996; Bruce et al., 1997; Hill et al., 1997). The classical form of BSE (also known as C-type BSE, to distinguish it from atypical forms) is therefore currently the only known zoonotic prion disease described.

Scrapie is a prion disease of sheep, endemic in many countries including the United Kingdom, which comprises several distinct strains (Greenlee, 2019). There is polymorphic variation in the sheep *PRNP* at amino acid positions 136, 154, and 171 that affects susceptibility to scrapie, and the VRQ, ARQ, and ARR genotypes have the greatest effect in this regard (Goldmann, 2008; Gonzalez et al., 2012). Depending on the infecting source or strain, VRQ/VRQ and ARQ/ARQ sheep are the most susceptible to classical scrapie, whereas ARR/ARR is effectively resistant (Hunter and Bossers, 2006). Although scrapie has been known to exist for more than 200 years, in stark contrast to BSE, there is no epidemiological evidence to suggest is poses a threat to human health (Brown, 1998; van Duijn et al., 1998).

A longstanding question is whether BSE can pose a zoonotic threat to humans via sheep. When considering zoonotic potential from animal prion diseases to humans as the target species, the risk posed by BSE from cattle as the species of origin is well known. It remains unknown whether the United Kingdom sheep flock was exposed to BSE early in the BSE epidemic, and whether BSE could propagate and adapt in sheep to resemble scrapie but retain its zoonotic potential for infecting humans. However, various experimental approaches have been taken to model this scenario as described below.

### BSE in Sheep

Although no natural cases of BSE in sheep have been detected, sheep can be experimentally infected with BSE (Foster et al., 2001), and it had been suggested that the clinical signs of scrapie and sheep-BSE are clinically indistinguishable (Stack et al., 2006) and that these forms of prion disease could only be differentiated by careful immunohistochemical examination and biochemical analysis of the protease resistant PrP^Sc^ in the central nervous system of affected animals (Jeffrey et al., 2001; Gonzalez et al., 2003; Thuring et al., 2004). It is therefore possible that adaptation of the BSE agent to an ovine host involves a change in the molecular properties of the BSE prions and that this change may have occurred prior to the introduction of statutory testing of sheep for scrapie. An important concern is whether sheep-adapted BSE would retain the potential to infect humans, or indeed be more virulent (Espinosa et al., 2007; Plinston et al., 2014).

### Using Serial PMCA to Assess the Zoonotic Risks

Many transmission studies have investigated the potential of BSE to infect sheep (Foster et al., 2001; Gonzalez et al., 2003; Stack et al., 2009), and for sustained transmission of BSE in flocks (Bellworthy et al., 2005; Jeffrey et al., 2015), and the potential for BSE-infected sheep to infect humans (Plinston et al., 2014; Joiner et al., 2018). As mentioned previously, a disadvantage of these *in vivo* investigations is that they are often lengthy and expensive.

**FIGURE 3:**
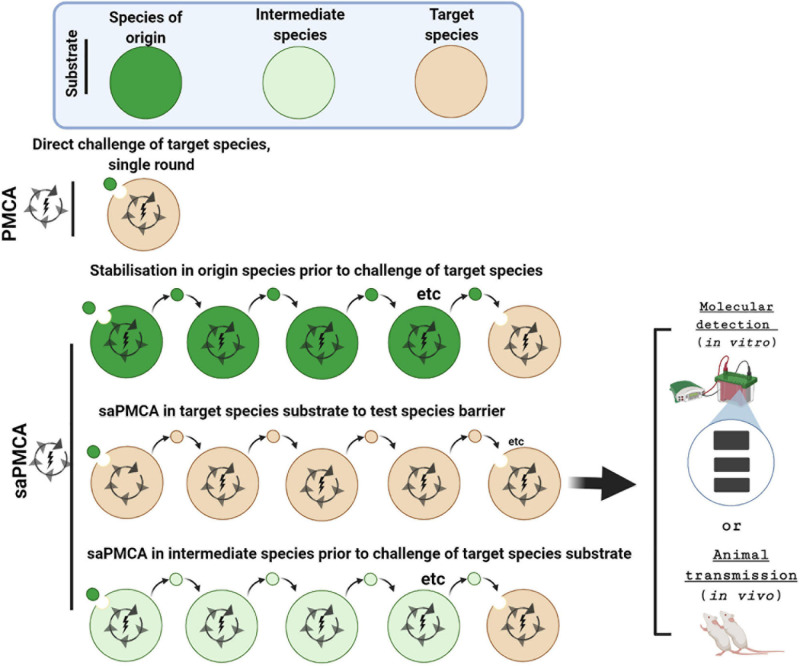
Investigating inter-species transmissibility by PMCA and saPMCA. The potential for a prion strain to cross the species barrier between the species of origin (dark green) and the target species (brown) can be assessed by a single round PMCA experiment. Alternatively, in serial automated PMCA (saPMCA), the amplification phase can be extended by multiple rounds in which the seed from one round is diluted into fresh PrP^C^ substrate. Multiple rounds of saPMCA using PrP^C^ from the species of origin might stabilize a prion strain to increase inter-species transmissibility. Alternatively, saPMCA with target species PrP^C^ could be used to examine the potential for a strain to adapt to a new replication environment. Finally, saPMCA can be used to address whether propagation with PrP^C^ from an intermediate species (light green) facilitates transmission to the target species.

The *in vitro* conversion assay, PMCA, provides an alternative means for investigating species barriers and for specifically examining the molecular feasibility of zoonotic transmissions. In the serial automated PMCA (saPMCA), the product from one round of PMCA is used to seed fresh PrP^C^ substrate in successive rounds of PMCA performed in series (Castilla et al., 2005, 2006) (Figure 3). By providing fresh PrP^C^ substrate with each round, saPMCA overcomes a major limitation of PMCA which is the thermolability of PrP^C^ at 37°C and thereby extends the amplification process. Therefore, saPMCA can be used to investigate whether the propagation of a prion strain with PrP^C^ substrate from another species is possible and moreover whether in can be sustained (Castilla et al., 2005; Krejciova et al., 2014). Serial automated PMCA can also be used to investigate whether the propagation of a prion strain from the species of origin via an intermediate species modifies its potential for infecting and propagating in the target species in question, for example humans.

### Investigating Whether a Prion Strain Can Be Continuously Propagated *in vitro*

The potential for BSE prions to be continuously propagated in sheep was investigated by saPMCA. Using sheep ARQ/ARQ PrP^C^ as a substrate, Krejciova et al. (2014) showed that BSE from cattle, or experimentally challenged sheep, could be continuously amplified over at least eight rounds. The PrP^Sc^ subtype was unaltered, suggesting a conservation of the BSE agent strain using this substrate. These findings agreed with an animal transmission study showing BSE prions could be serially passaged three times through sheep of the ARQ/ARQ genotype, with only minor alternations of the molecular properties of PrP^Sc^ (Stack et al., 2009). In contrast, PrP^C^ from ARR/ARR sheep, a genotype known to be more resistant to scrapie, could not sustain the amplification of cattle or sheep BSE PrP^Sc^ over successive rounds (Krejciova et al., 2014), even though amplification of sheep BSE PrP^Sc^ was observed for seeds in the first round.

### Modeling Strain Adaption

In addition to testing for sustainable prion propagation, saPMCA can also be used to investigate whether the properties of the strain are modified and to explore the possibility of strain adaption in either an intermediate species or the target species. Alterations in the molecular profile of the PrP^Sc^ amplified product can be used as indicator of changes to agent strain (Castilla et al., 2008a). In most cases these will be changes in the molecular mass of the unglycosylated fragment or the relative intensities of the di-, mono- and unglycosylated fragments, which can be monitored by analyzing samples of the saPMCA rounds by western blotting.

Changes in the molecular subtype that arise during saPMCA can also be detected using PrP^Sc^ subtype-specific antibodies. For instance, when sheep BSE was propagated using ovine VRQ/VRQ substrate by saPMCA, the generic anti-PrP 6H4 monoclonal antibody detected an upward shift to more scrapie-like molecular profile in the latter rounds. This was further verified using the 12B2 monoclonal antibody, which specifically detects the longer, slower migrating protease resistant PrP^Sc^ fragment associated with scrapie (Krejciova et al., 2014). This indicates a possible strain adaptation that might occur *in vivo* if BSE infection spread into a sheep population.

This was not the first study to show that saPMCA could model the process of strain adaptation. For instance, Green et al. (2008) demonstrated that saPMCA could enable mouse prions of the RML strain to adapt to replication using deer PrP^C^. When RML prions were propagated using cervid PrP^C^ expressed in TgCer mice the physiochemical properties and western blot profile of PrP^Sc^ were altered, and there was an increased ability to infect TgCer mice resulting in a prion strain with unique properties (Green et al., 2008).

### Mechanisms of Strain Adaption

The mechanism by which prion strain adaptation occurs *in vitro* or *in vivo* is still at matter of debate. The shift in the molecular profile observed after multiple rounds of saPMCA using PrP^C^ from a different species as substrate is compatible with the idea that the strain of origin, such as BSE, might comprise a molecular cloud of conformers. When a prion strain is introduced into the target or intermediate species, a subset of conformers is selected for amplification (Collinge and Clarke, 2007; Krejciova et al., 2014; Collinge, 2016). In this model, if a large subset of conformers of the original prion strain can replicate in the target species, the species barrier will be low. The size of the subset of conformers that can replicate in both species will partly be determined by the primary structure of PrP from both species.

An alternative but not mutually exclusive model is described as “deformed templating.” In this model, when a prion strain is introduced into a new species, PrP primary structural differences between the species impose molecular constraints that force the generation of a range of new PrP^Sc^ variants (Makarava and Baskakov, 2012; Makarava et al., 2013; Baskakov, 2014). One of these variants may prove to be particularly adept at replication in the new host, resulting in the emergence of a new strain. With either model, PMCA and saPMCA are capable of modeling strain adaptation, regardless of the mechanism.

The conformational selection or deformed templating models are consistent with the findings of Huor et al. (2019) who used both animal transmission studies and saPMCA to show evidence of a C-type BSE-like signature emerging from atypical scrapie. When atypical scrapie PrP^Sc^ seeds were amplified over several rounds of saPMCA using TgBov substrate, PrP^Sc^ with a BSE-like molecular profile was amplified, that could be clearly discriminated from scrapie-like PrP^Sc^ using PrP^Sc^ subtype specific antibodies. These results indicated the theoretical possibility that classic BSE might arise through cross-infection from atypical sheep scrapie. In this way PMCA can amplify subsets of these molecular clouds that point to the existence of minority strains that may exist within other strains. In this way, PMCA and saPMCA may offer insights into the provenance of certain prion strains.

### Zoonotic Threat After Strain Adaptation in an Intermediate Species

An advantage of saPMCA is that amplification protocols can be concatenated, i.e., the end-product of one amplification protocol can be used to seed further amplification using substrate from an individual with a different genotype, or another species entirely (Figure 3). This approach is useful for investigating whether an animal prion disease becomes a threat to human health after cross-infection and propagation in an intermediate species. Humanized transgenic mouse brain (TgHu) expressing the three polymorphic genotypes of *PRNP* codon 129 (TgHuMM, TgHuMV, TgHuVV) have been commonly used as a PMCA substrate to examine the zoonotic threat posed by animal prion diseases (Barria et al., 2014b).

The species-specificity of certain anti-PrP antibodies has been used to evaluate the effect of *in vitro* studies of zoonotic potential. These antibodies specifically detect the conversion of the substrate PrP^C^ to protease resistant PrP^Sc^ without interference from the small amount of PrP^Sc^ that was added as seed. For example, 3F4 recognizes the 106-112 epitope in human, but not ovine or bovine PrP.

Krejciova et al. (2014) used 3F4 to show that C-type BSE from cattle or experimentally infected sheep could induce the conversion of human PrP^C^ to PrP^Sc^ in single-round PMCA. BSE PrP^Sc^ that had retained its BSE-like molecular signature after amplification using an ARQ/ARQ ovine substrate also retained its ability to convert human PrP^C^. In both cases, conversion was dependent on the codon 129 genotype of human PrP being MM. However, BSE PrP^Sc^ that had adapted and acquired a scrapie-like molecular signature after amplification using VRQ/VRQ substrate had lost its ability to convert human PrP^C^. This experimental observation may suggest that BSE PrP^Sc^ can adapt and adopt the guise of a scrapie-like strain in sheep, but this adaptation reduces its zoonotic threat to humans.

The effects of the adaption of BSE PrP^Sc^ to sheep on its virulence toward humans have also been analyzed by animal transmission studies. Padilla et al., 2011 showed that BSE prions from cattle could infect TgHu mice that over-express PrP^C^ MM at codon 129, albeit with a large transmission barrier. This barrier was significantly reduced when the same mouse models were challenged with PrP^Sc^ from BSE-infected sheep and goats suggesting an increased zoonotic threat from BSE in these species (Padilla et al., 2011). However, it is not known what effect adaptation of BSE prions to sheep or goats by multiple passages would have on its virulence toward humans.

### Emergent and Atypical Animal Prion Diseases

#### Assessing the Zoonotic Threat of CWD and Atypical Forms of Scrapie and BSE

The zoonotic threat to humans of BSE in cattle is well known. In contrast, there is no epidemiological evidence to support scrapie in sheep posing a risk to human health (Brown et al., 1987; van Duijn et al., 1998). The zoonotic threat of CWD in cervids, and atypical forms of both scrapie and BSE are unknown. However, a number of *in vitro* and transmission studies using TgHu mice or non-human primates suggest a significant barrier for CWD transmission to humans [see Table in Barria et al. (2014a) for a summary of the *in vitro* and *in vivo* approaches that have been used]. Furthermore, challenge of TgHu mice with atypical H- and L-type BSE, showed that L-type BSE is capable of transmitting disease to transgenic mice overexpressing a human PrP (Beringue et al., 2008).

Transmission studies using mice expressing human PrP, or non-human primates, provide a rigorous assessment of the zoonotic threat to humans posed by animal prion diseases (Brandner and Jaunmuktane, 2017). However, this approach is often slow and expensive. In contrast, *in vitro* conversion assays such as PMCA are fast and adaptable. They can be used to assess the potential for the heterologous conversion of human PrP by newly emerging animal prion diseases, or atypical forms of classic animal prion diseases. The approach of testing seeds from multiple species in parallel against substrate from one species (e.g., human) allows the zoonotic potential of several prion strains to be compared. The results of these experiments can be judged alongside evidence from epidemiology and transmission studies.

An interesting observation of many of these comparisons is the concept of conversion efficiency as a possible measure of zoonotic potential, which can be inferred from the levels of PrP^Sc^ that have accumulated after a defined period of amplification. In this regard, the reliability of PMCA for modeling transmission barriers has been evaluated by Levavasseur et al. (2014). Using vCJD and atypical BSE seeds against various transgenic mouse brain substrates, a good agreement between the amplification factors obtained by PMCA results and attack rates for parallel *in vivo* transmission experiments was observed (Levavasseur et al., 2014).

Chronic wasting disease is an acquired prion disease of cervids that affects captive and free-ranging deer and elk populations primarily in North America but recently reported in Europe (Sigurdson and Aguzzi, 2007; Benestad et al., 2016). Recently, PMCA has been used to study strain adaptation of CWD prions to a range of cervid host PrP^C^ sequences, suggesting there could be a constantly evolving diversity of CWD conformers. This has implications for the potential emergence of CWD in new Cervid species (Duque Velasquez et al., 2020) that may in turn pose a threat to human health.

Protein misfolding cyclic amplification has been used to assess the molecular compatibility and the direct zoonotic threat that CWD might pose to humans. Barria et al., 2011 reported that CWD PrP^Sc^ from mule deer (*Odocoileus hemionus*) could convert human PrP from TgHu substrate to produce PrP^Sc^ with a unique molecular profile, contrasting to the observations reported by Kurt et al. (2009, 2015) of a significant species barrier between humans and cervids; However, extensive *in vitro* conditioning of the mule deer CWD isolate (using cervid PrP^C^ substrate in PMCA or serial transmission in a cervidised transgenic mouse model) was necessary to overcome the molecular barrier and support the conversion of the human PrP^C^ (Kurt et al., 2009, 2015; Barria et al., 2011).

In a later study, Barria et al., 2014a used PMCA to address the possibility of direct zoonotic transmission of prions from cervids to humans, and compared the results with scrapie and BSE prions. Using PrP^C^ substrate from human, TgHu mouse brain and 293F cultured cells, we showed that CWD PrP^Sc^ from North American elk (*Cervus canadensis*) could convert PrP^C^, albeit less efficiently than BSE (Barria et al., 2014a). This study concluded that there were no absolute molecular barriers for CWD to infect humans.

In addition, the zoonotic potential of CWD was further examined by testing prion seeds from North American elk of different cervid *PRNP* codon 132 genotypes and wild-tailed deer (*O. virginianus*), against TgHu substrate in a single-round PMCA. All cervid seeds induced conversion of human PrP^C^, although conversion efficiency depended on the compatibility of the codon 132 genotype of cervid PrP and the corresponding codon 129 of human *PRNP* (Barria et al., 2018b).

#### Zoonotic Threat of Atypical Animal Prions

Atypical scrapie, also known as Nor98, is a rare prion disease of sheep identified as a result of active surveillance for scrapie (Benestad et al., 2003). In PMCA investigations of its zoonotic potential, Nor98 failed to induce significant conversion using PrP^C^ substrate from human, TgHu mouse brain and 293 F cultured cells in contrast to BSE and human CJD positive controls (Barria et al., 2014a).

Atypical BSE is a rare, atypical prion disease of cattle comprising two forms: L-type BSE and H-type BSE. L-type BSE, also known as bovine amyloidotic spongiform encephalopathy, is neuropathologically characterized by amyloid plaques in the brain, and has a PrP^Sc^ subtype similar to type 2A in human sCJD patients, with a faster migrating unglycosylated fragment compared with C-type BSE (Casalone et al., 2004). The other atypical form, H-type BSE, is characterized by a slower migrating protease resistant PrP^Sc^ fragments compared with C-type BSE (Biacabe et al., 2004). In contrast to C-type BSE which is acquired, L-type and H-type BSE are believed to be sporadic.

Protein misfolding cyclic amplification was used to assess the potential for atypical BSE to cross the species barrier to humans. Neither L-type nor H-type BSE induced significant conversion in PMCA using PrP^C^ substrate from human, TgHu mouse brain and 293 F cultured cells (Barria et al., 2014a, b), in contrast to C-type BSE and human CJD positive controls (Barria et al., 2014a). This could be taken to suggest that the atypical forms of BSE pose a poor zoonotic threat, or are far less likely to transmit to humans compared to classic BSE. However, an earlier finding reported that L-type BSE was able to produce infectivity in a humanized transgenic mice model overexpressing the human prion protein (Beringue et al., 2008).

#### Zoonotic Threat of CWD Emerging in Europe

The first identification of CWD in Europe occurred in 2016 in wild moose (*Acles alces*, also known as Eurasian elk) and in a free-ranging reindeer (*Rangifer tarandus*, closely related to the free-ranging caribou of North America), a species not previously known to be affected by CWD in wild or farmed animals (Benestad et al., 2016). The molecular compatibility and zoonotic potential of CWD in reindeer was examined by testing prion seeds from two reindeer experimentally infected with CWD from white-tailed deer (Mitchell et al., 2012). Seeds prepared from these reindeer brains were tested against TgHuMM, TgHuMV, TgHuVV substrates in a single-round PMCA (Barria et al., 2018b). This study again used the 3F4 monoclonal antibody to detect the induced conversion of human PrP^C^ to protease resistant PrP^Sc^. Samples of PrP^Sc^ from one of the two CWD infected reindeer could induce robust conversion of MM, MV, and VV substrates. The other reindeer had a lower abundance of PrP^Sc^ and showed weaker conversion of all three substrate genotypes tested. Barria et al., 2018b showed that low levels of experimentally infected reindeer brain PrP^Sc^ could be partially purified and normalized (according to the level of protease-resistant PrP^Sc^) and used to successfully seed PMCA reactions. The influence of codon 129 on the molecular barriers for conversion of human PrP^C^ by reindeer PrP^Sc^ is currently being further investigated. Consistent with our previous findings and those reported previously by Raymond et al. (1997, 2000) and (Barria et al., 2011, 2014a, b, 2018b), we concluded that there were no absolute molecular barriers to the conversion of human PrP^C^ by CWD PrP^Sc^, and a more comprehensive and thorough assessment of the zoonotic potential of CWD might be needed.

### Investigating the Potential of CWD to Infect Other Non-human Species

The above PMCA studies involved comparing seeds from multiple species against substrate from one species, namely humans. An alternative approach is to test one seed on substrates from multiple species. For example, this approach can be used to assess the threat posed by CWD infected cervids to other species that share its environment in the wild. Furthermore, PrP sequence alignment can be used to assess the key determinants of the species barrier. Kurt et al., 2009 used saPMCA to show that vole and field mouse PrP^C^ supported serial amplification of white tailed deer (WTD) CWD PrP^Sc^, whereas prairie dog and coyote PrP^C^ did not. Sequence comparisons of multiple species substrates showed that asparagine at codon 170 of the substrate PrP^C^ was important for supporting trans-species conversion (Kurt et al., 2009).

**FIGURE 4:**
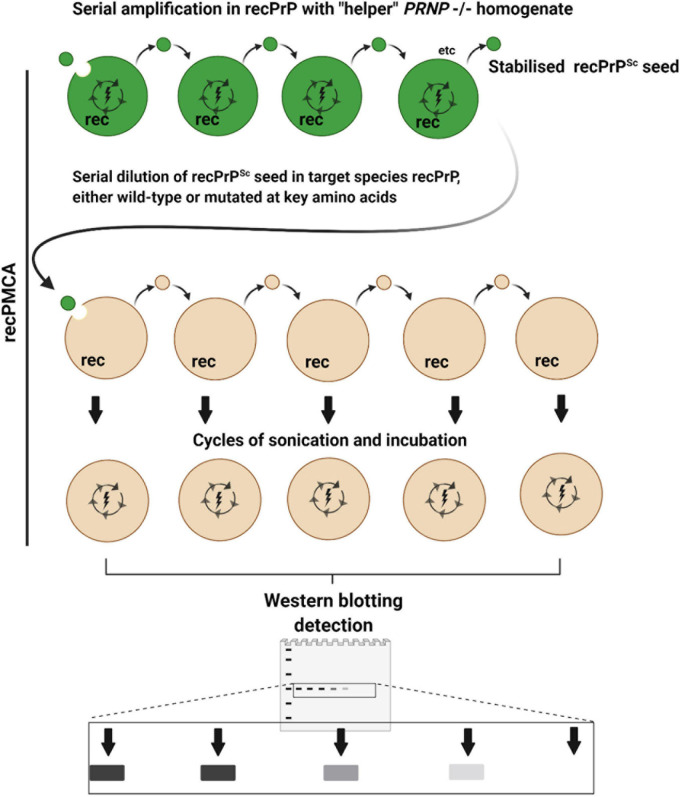
Recombinant PMCA (recPMCA). In recPMCA, bacterially expressed and purified recombinant PrP (recPrP) is used as a substrate for conversion. Firstly, PrP^Sc^ from infected tissue of the species of origin (dark green) is propagated by saPMCA using recPrP from the same species to produce recPrP^Sc^ as an end product, detectable on western blots as a single band. Propagation in recPMCA requires the presence of “helper” brain homogenate from *PRNP*–/– animals. Thus, the effects of variable PrP^C^ expression, glycosylation and cofactors that affect conventional PMCA reactions are largely eliminated, to focus primarily on the effect of sequence and conformational compatibility. The levels of recPrP^Sc^ are normalized and the recPrP^Sc^ is serially diluted in recPrP from the target species before being subjected to cycles of sonication and incubation. Species barriers are assessed according to how abruptly PMCA conversion is diminished by serial dilution of the seed: i.e., if the barrier is low, highly diluted seed will still cause conversion. Mutant forms of recPrP can easily be generated to examine the effect of amino acid substitutions on species barriers.

The potential for cross transmission of prion disease between cervids and sheep has been investigated using another version of the PMCA concept, namely recPMCA (Figure 4). This method is a variation of saPMCA that focuses on the effect of PrP sequence differences on molecular compatibility (Erana et al., 2017; Fernandez-Borges et al., 2017). When recPMCA is used to investigate species barriers, the seeds from the species of origin are firstly amplified in multiple rounds of PMCA using bacterially expressed recombinant PrP (recPrP) of the same species. Although unglycosylated, it is assumed that the recPrP^Sc^ end products retain some of the conformational properties of the original seed. The levels of the recPrP^Sc^ seeds are normalized, before being serial diluted and used to seed PMCA reactions containing wild-type or mutant recPrP from one or more target species (Erana et al., 2017; Harrathi et al., 2019). The advantage of using recPrP as a substrate is that mutant forms can be readily engineered to investigate the effects of amino acid substitutions, deletions, or insertions.

Using recPMCA Harrathi et al. (2019) were unable to serially convert wild-type sheep recPrP substrate using CWD recPrP^Sc^, indicating a significant species barrier for the transmission of CWD to sheep. However, single residue substitutions at four sites in ovine PrP affected its potential to be converted by CWD recPrP^Sc^, which offered insights into PrP sequence-structure factors affecting the potential for cross-infection from cervids to sheep, such as the β2-α2 loop (Harrathi et al., 2019). It should be noted that transmission studies where sheep have been challenged with mule deer CWD, and elk challenged with sheep scrapie, suggest that the species barrier between sheep and cervids is not absolute (Hamir et al., 2004; Cassmann et al., 2021).

### Advantages and Limitations of saPMCA for Assessing Zoonotic Potential

For prion diseases, some of the factors linked to the species barrier phenomenon include (1) differences between the *PRNP* sequences of the species (2) the animal prion strain, as enciphered in the conformation of PrP^Sc^, and (3) the physiological differences between humans and the animal in question. The effect of cofactors on prion conversion and the glycosylation of PrP^C^ are two other species-specific phenomena that contribute to the species barrier. PMCA can model aspects associated with the molecular and subcellular compatibility of the PrP^Sc^ structure and PrP sequence, previously referred to as the “sequence – structure barrier” (Baskakov, 2014). Although PMCA may be limited in its ability to model additional factors operating above the subcellular level, it provides a powerful method for investigating the features affecting the molecular compatibility between heterologous PrP^Sc^ and PrP^C^ that are still not fully understood. The fact that PMCA produces bona fide PrP^Sc^ as an end product means that PMCA can help to indicate the molecular profile that may arise from a cross-species infection. The properties of the PrP^Sc^ end product can be assessed, such as the western blot profile, or its equilibrium dissociation curve following denaturation with increasing concentrations of guanidine HCl (Yokoyama et al., 2011). Furthermore, this product can be used in onward animal transmission studies, to obtain additional strain characteristics including clinical signs, the neuroanatomical distribution of lesions and PrP^Sc^ deposition and incubation periods (Beck et al., 2013).

A practical advantage of PMCA for modeling species barriers is speed. A single round of PMCA typically takes 48 h, although multiple rounds of saPMCA may take several weeks. However, this is considerably shorter that the months or years taken to complete animal transmission studies.

An important consideration in PMCA is ensuring the availability and consistency of the substrate. Conversion is most efficient and reliable using perfused brain from experimental animals, usually transgenic mice. For assessing the zoonotic threat to humans, post-mortem human brain tissue with consent for use can be used, but there are factors that confound conversion efficiency and consistency, including post mortem interval, the presence of blood, and variability between samples in terms of the relative content of gray and white matter. To improve consistency, the use of a stably transfected human cell cultures as a source of human PrP^C^ substrate for PMCA has been demonstrated (Yokoyama et al., 2011; Barria et al., 2014a, b).

The drive toward more efficient PMCA can have its own counterpoint. Some particular observations suggest we should be cautious when modeling species or genotypic barriers with PMCA. For instance, Vidal et al. (2013) were able to amplify BSE PrP^Sc^ by saPMCA using PrP^C^ from rabbits and dogs, even though the latter species are considered resistant to prion disease. Lacroux et al. (2014) have developed a PMCA assay using ovine Q171 substrate which can efficiently amplify BSE/vCJD prions regardless of the species of origin. Therefore, with their methodology, PrP sequence homology between seed and substrate does not appear to be crucial for BSE/vCJD prion replication *in vitro* (Lacroux et al., 2014). It has also been shown that unseeded saPMCA using leporid PrP^C^ substrate could lead to the *de novo* formation of PrP^Sc^, which could induce disease when used to inoculate rabbits (Chianini et al., 2012). This suggests that increases in the efficiency of PMCA can circumvent species barriers, and induce disease in species that were thought to be resistant, and therefore careful interpretation of the results of PMCA experiments is required.

Even when a prion strain is known to be zoonotic, PMCA could potentially underestimate the species barrier. For instance, several PMCA studies have shown that human PrP^C^ (M at codon 129) is readily converted by BSE PrP^Sc^ in a manner dependent upon the genotype at *PRNP* codon 129 (Raymond et al., 1997; Jones et al., 2009; Barria et al., 2014a; Krejciova et al., 2014). However, animal transmission studies pointed to a higher transmission barrier for BSE between cattle and humans. Transmission of cattle BSE to TgHu mice overexpressing human PrP^C^ has been shown to be inefficient (Asante et al., 2002; Padilla et al., 2011), and no transmission of cattle BSE was observed using an alternative gene-targeted TgHu mouse model (Bishop et al., 2006). It should be noted that the extent of the vCJD epidemic was relatively small compared with the very high numbers cattle that were infected with BSE. Therefore, the complementation of *in vitro* and *in vivo* models are important to understand the complexity surrounding species barrier and the zoonotic potential to humans.

## Conclusion

Where as animal transmission studies are an effective means for investigating species barriers, PMCA continues to provide a time- and cost-efficient alternative that can offer genuine insights into the molecular feasibility of cross-species transmission, and some of the strain properties of PrP^Sc^ that may arise. PMCA can be used to assess whether prion strains can transcend species barriers, and also genotypic barriers within the same species. Unlike other *in vitro* conversion assays, PMCA can predict alterations in the molecular profile of PrP^Sc^ that might occur upon adaptation to a new species. PMCA can also uncover evidence of traceback, a give indications on the likely origin of a prion strain. Also, PMCA, using substrate from the target species, can be used to model the molecular compatibility and zoonotic risk of the prion agent. In summary, the PMCA group of techniques provide a powerful approach for assessing intra- and inter-species barriers, focused on the molecular feasibility of prion conversion. However, the results should be interpreted with caution, and can be complemented with animal transmission and epidemiological studies in circumstances where they are available.

## Author Contributions

AP, SS, and MB: writing—original draft preparation, review and editing. MB: conceptualization. All authors: read and agreed to the published version of the manuscript.

## Author Disclaimer

The views expressed in this publication are those of the author(s) and not necessarily those of the Department of Health and Social Care or the Government of Scotland.

## Conflict of Interest

The authors declare that the research was conducted in the absence of any commercial or financial relationships that could be construed as a potential conflict of interest.

## Publisher’s Note

All claims expressed in this article are solely those of the authors and do not necessarily represent those of their affiliated organizations, or those of the publisher, the editors and the reviewers. Any product that may be evaluated in this article, or claim that may be made by its manufacturer, is not guaranteed or endorsed by the publisher.
